# How followers’ perception of followership prototype–traits fit impacts their job performance: a moderated polynomial regression analysis

**DOI:** 10.3389/fpsyg.2023.1279568

**Published:** 2024-01-08

**Authors:** Jing Yu, Jian Feng

**Affiliations:** ^1^School of Business Administration, Southwestern University of Finance and Economics, Chengdu, China; ^2^Student Career Planning and Employment Guidance Center, Southwestern University of Finance and Economics, Chengdu, China

**Keywords:** followership prototype, followership traits, taking charge, job performance, followership

## Abstract

**Introduction:**

Previous studies about the drivers of follower performance focused on leadership, and most followership studies have used a single perspective to investigate this topic from the followers’ lens. Therefore, the purpose of this study is to explore *whether*, *how*, and *when* followers’ perception of followership prototype–traits fit influences their job performance.

**Methods:**

The study adopted a questionnaire survey (Study 1) and a scenario experiment (Study 2). First, in the questionnaire survey, we collected 72 leaders and 262 followers from 72 teams of 14 companies in China using a two–wave research design. Second, in the scenario experiment, we invited 160 undergraduates from a university in southwest China to participate in the experiment after verifying the effectiveness of the manipulated materials.

**Results:**

(1) compared with the misfit, followership prototype–traits fit is more likely to stimulate followers’ taking charge; (2) compared with low levels of fit, high-level followership prototype–traits fit is more likely to stimulate followers’ taking charge; (3) compared with high followership prototype and low followership traits condition, low followership prototype and high followership traits condition is more likely to stimulate followers’ taking charge; (4) followers’ taking charge mediates the impact of the followership prototype–traits fit on followers’ job performance; and (5) the impact of followership prototype–traits fit on followers’ taking charge is more salient for male followers than for female followers.

**Discussion:**

This study not only helps capture the bidirectional and complex process of the interaction between leaders and followers during the followership, but also obtains a more comprehensive understanding of how this interaction affects followers’ behaviors and performance. The results have practical implications for improving followers’ job performance by highlighting the effects of followership prototype–traits fit on followers’ behaviors and performance.

## 1 Introduction

Almost everyone is a follower in the workplace. It is crucial to improve followers’ job performance to enable the organization’s survival and thrive in today’s VUCA environment. Therefore, many scholars have devoted a great deal of attention to investigating the drivers of followers’ job performance. Unfortunately, these studies have largely focused on the leadership lens, such as the leader’s traits and behaviors (Chen et al., 2007; Den Hartog and Belschak, 2012). Uhl-Bien et al. (2014) pointed out that there will be no leaders or leadership without followers. Since then, research on how followership impacts followers’ job performance has been gaining ground.

Until now, followership research enjoys a variety of theories and concepts (Uhl-Bien et al., 2014). We conducted a brief review of theoretical perspectives in followership studies, categorizing them into four aspects. The first theoretical perspective is *trait perspective*, which focuses on the influence of follower traits on leadership styles, behaviors, effectiveness, and the relationship with leaders. The second is *motivational perspective*, which focuses on uncovering followers’ motivations behind different types of followership, such as to satisfy their needs (Kellerman, 2008) and obtain valuable resource (Derue and Ashford, 2010). Essentially, this line of inquiry addresses the question of “why follow.” The third is *behavioral perspective*, which focuses on the specific behaviors displayed by followers in the followership process, exploring “how to follow” (Brumm and Drury, 2013; Wang et al., 2022). The fourth is *cognitive perspective*, which revolves around implicit followership theories (IFTs). IFTs are defined as “leaders’ personal assumptions about the traits and behaviors that characterize followers” (Sy, 2010, p. 73). Extant scholars have utilized this theoretical lens to conduct a series of studies from a congruence perspective, such as exploring the impact of leader–follower followership prototype congruence effects on follower outcomes (Peng and Wang, 2016; Wang and Peng, 2016; Peng and Wang, 2018).

It is worth note that extant followership studies have largely used a single followers’ perspective to conduct investigation, such as studies based on trait, motivational, and behavioral perspectives (Benson et al., 2016; Wee et al., 2017). However, Kim et al. (2020) noted that leadership and followership develop together. Studies from a single perspective may lead to an oversimplified understanding of the followership process and even erroneous conclusions (Foti et al., 2017; Wu et al., 2021). Therefore, adopting a leader–follower dyadic perspective based on *cognitive perspective* is necessary to explore the antecedents of follower performance. Some studies have also employed this dyadic perspective to explore the impact of leader–follower congruence regarding personality or values on followers’ job performance (Zhang et al., 2012; Carter and Mossholder, 2015), which helps advance our understanding of the drivers of followers’ job performance.

Additionally, leader–follower interaction is substantially influenced by leader’s IFTs as IFTs capture leaders’ idiosyncratic perspectives on what constitutes appropriate followership (Graen and Scandura, 1987; Sy, 2010; Epitropaki et al., 2013; Junker and van Dick, 2014). The followership prototype captures leaders’ overall expectations for followers’ traits, which is the core component of IFTs (Sy, 2010). Additionally, the extent to which leaders view unique followers’ traits favorably depends on whether these traits align with the leaders’ followership prototype (Epitropaki et al., 2013; Peng and Wang, 2018). For instance, a follower’s proactive personality may be perceived as beneficial by a leader who values proactivity but might be seen as a threat by a leader expecting followers to strictly adhere to orders and instructions (Zhang et al., 2012). Therefore, the (mis)alignment between leaders’ followership prototype and followers’ actual traits determines the effectiveness of followership, thereby influencing followers’ behaviors and performance (Peng and Wang, 2018). Therefore, we thus focus on the influence of the fit between leaders’ followership prototype and followers’ followership traits (referred to as “prototype” and “traits,” respectively, hereafter) on followers’ job performance. In doing so, we contribute to a more comprehensive understanding of the ramifications of prototype-traits fit in the followership literature.

Since polynomial regression and response surface analysis can help clarify the complex influence of leader–follower interaction on followers from the dyadic perspective of leaders and followers (Lam et al., 2018; Stegmann et al., 2020), we use this method to explore *whether, how*, and *when* perceptions of followership prototype–traits fit impacts followers’ job performance. By doing so, we further advance the knowledge about the relationship between leader–follower congruence and follower performance and inform business practices.^1^

Based on the COR theory, we investigate how prototype–traits fit affects followers’ job performance. The core tenet of COR theory posits that individuals strive to obtain, retain, and protect valuable resources, and the total amount of resources they own affects their behavior and performance (Hobfoll, 2001; Hobfoll et al., 2018). According to COR theory’s resource caravan principle, resources do not exist in isolation but work in packs or caravans (Hobfoll et al., 2018). For example, an individual’s internal characteristic resources and resources obtained from external sources (such as leaders) constitute an individual’s total resources. Followership traits are valuable characteristics of followers, and leaders will provide differentiated external resources according to whether the followers fit their prototype. Therefore, prototype–traits fit will determine the total work resources allocated to the followers.

Furthermore, COR theory reveals that followers who obtain resources from followership prototype–traits fit tend to reinvest the resources into the workplace by conducting desirable work behaviors (Hobfoll, 2001). Fuller et al. (2006, pp. 1095–1096) further posited that those followers “*will engage in constructive change-orientated behavior and utilize resources to solve problems, experiment, make work-related improvements, and take advantage of new opportunities*.” Taking charge refers to constructive behavior that individuals voluntarily initiate to achieve organizational or functional changes, including optimizing operational processes and introducing innovative work methods (Morrison and Phelps, 1999). Research has suggested that taking charge is a crucial leader-valued extra-role behavior as it substantially enhances team and organizational effectiveness (Morrison and Phelps, 1999; Kim et al., 2015; Xu et al., 2023). Thus, followers’ taking charge plays a critical role in shaping the leader–follower relationship (Parker and Collins, 2010). Taking these arguments together, we predict that when followers obtain more resources due to their traits aligning with their leader’s prototype, they tend to initiate taking charge that the leader values to reciprocate their leader. Conversely, followers obtain fewer resources from the leader when their traits do not fit the prototype. According to COR theory, individuals with insufficient resources are more sensitive to resource loss and are less likely to take risky and proactive behaviors such as taking charge (Hobfoll et al., 2018).

Furthermore, followers who take charge tend to be motivated to improve their work and, therefore, significantly advance their job performance (Kim et al., 2015; Wu et al., 2018). In summary, by drawing on COR theory, we suggest that followers with different combinations of traits and prototypes have differentiated resources, which will impact the extent of the follower’s taking charge and the subsequent job performance.

**Figure 1:**
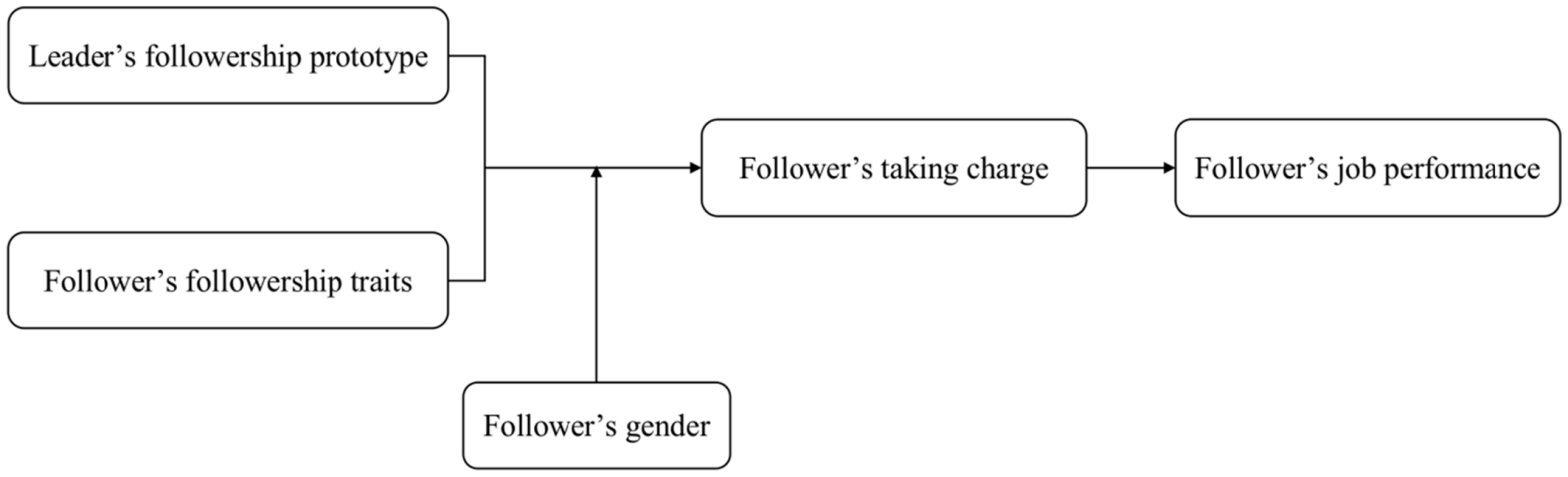
Theoretical model.

In addition, we argue that the impact of the prototype–traits fit on followers taking charge can also be moderated by social expectations of different genders (Eagly and Karau, 2002). Previous research has demonstrated that behavioral differences between men and women in their workplace responses to the same stimuli can be attributed to varying societal expectations regarding gender roles (Eagly and Karau, 2002; Eagly et al., 2020; Uzman et al., 2022). In our context, when confronted with a followership prototype–traits congruence, male and female followers will exhibit distinct behavioral responses shaped by their respective societal roles. Specifically, men are expected to be confident, ambitious, and achievement-oriented, while women are expected to be gentle, inclusive, and relationship-oriented (Eagly and Karau, 2002; Eagly et al., 2020). Such differentiated social expectations will lead to different probabilities of female and male followers’ engagement in taking charge to react upon followership prototype–traits fit (Luksyte et al., 2022). Therefore, this study proposed the research model illustrated in Figure 1 and explained below.

By employing two studies (i.e., a time-lagged survey and a scenario-based experiment) to test our theoretical model, our research provides several theoretical contributions. First, by exploring the impact of leader–follower congruence on followers’ job performance with the dyadic perspective, we not only advance the knowledge of antecedents of follower job performance but also help obtain a more comprehensive understanding of how leader–follower congruence impacts followers’ job performance. Second, we adopt a novel theory (i.e., COR theory) to reveal the mediating role of taking charge in transmitting the impact of perceived followership prototype–traits fit on followers’ job performance. Furthermore, by exploring the congruence effect between leaders’ followership prototype and followers’ followership traits on followers’ taking charge, we also help obtain a more comprehensive understanding of the antecedents of taking charge. Third, we highlight follower gender as a critical boundary condition is another notable contribution. Specifically, we found that male and female followers exhibit different preferences in employing taking charge when reacting to followership prototype–traits fit. This not only helps obtain a nuanced understanding of the congruence effect between followership prototype and traits on followers’ job outcomes but also enriches the boundaries about modifying the influences of leader–follower congruence on employees’ reactions.

## 2 Theory and hypotheses

### 2.1 COR theory and followership prototype–traits fit

COR theory posits that individuals strive to obtain, retain, and protect valuable resources to avoid loss (Hobfoll, 2001; Hobfoll et al., 2018). According to Hobfoll et al. (2018), resources are things that individuals consider valuable to them or the ways that may help them obtain valuable things, including materials (such as work environment), conditions (such as seniority), characteristics (such as self-efficacy), and energy (such as time, energy, and social support). According to the resource caravan principle of COR theory, an individual’s internal characteristics plus resources obtained from the external environment constitute that individual’s total resources (Hobfoll et al., 2018).

**Figure 2:**
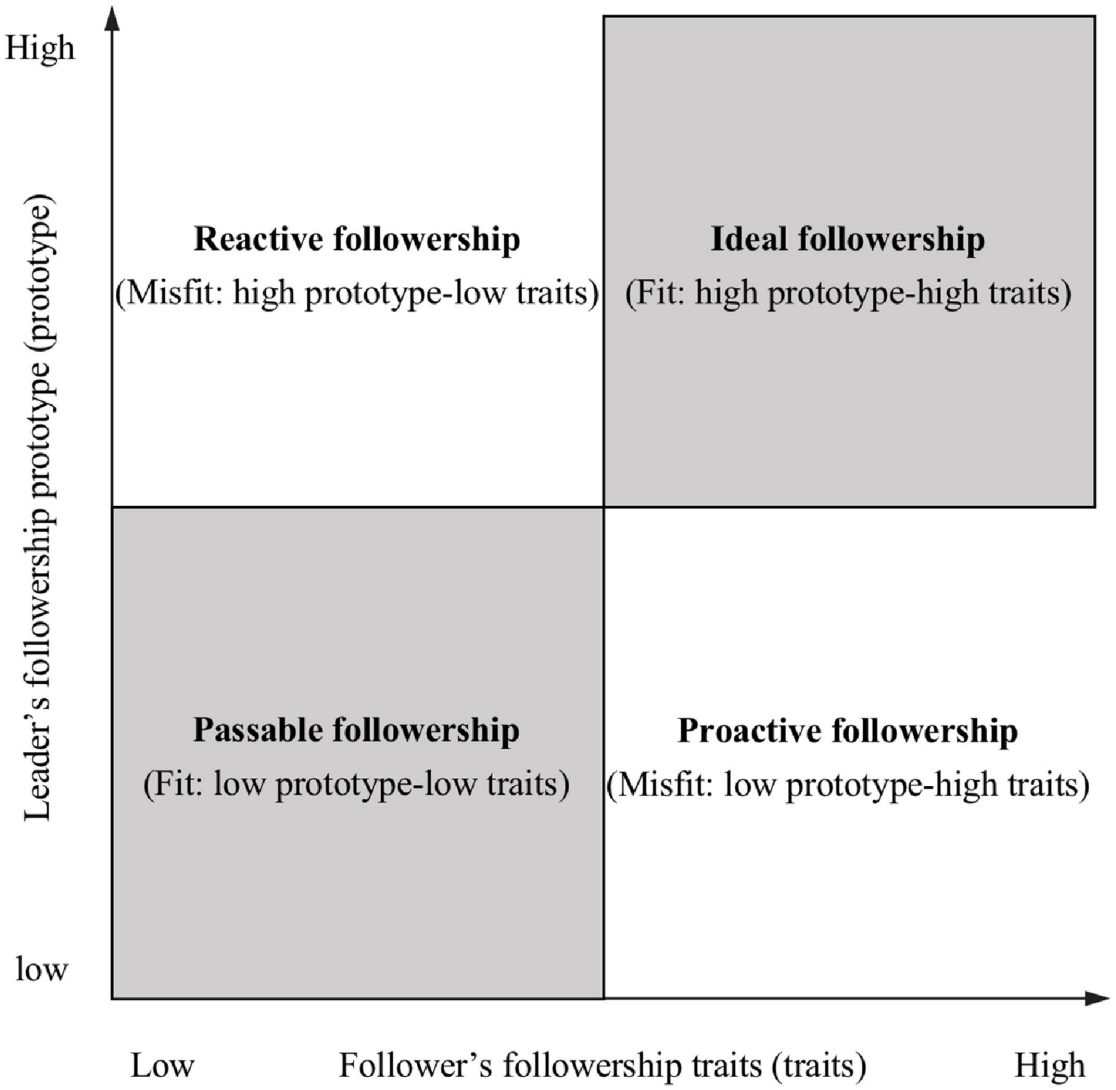
Four combinations of followership prototype and followership traits.

In this study, followership traits included industry (hardworking, productive, and goes above and beyond), enthusiasm (excited, outgoing, and happy), and good citizen (loyal, reliable, and team player) demonstrated by followers during the followership process (Sy, 2010). These positive traits are essential and valuable characteristics of followers (Sy, 2010; Hobfoll et al., 2018). Meanwhile, leaders will provide differentiated resources (such as instrumental and emotional support) based on whether the followers meet their leader’s prototype (van Gils et al., 2010; Peng and Wang, 2018). Thus, we propose that the fit between followership traits and followership prototype affects the number of resources followers can access at work. According to the level of followership prototype–traits fit, four followership types are formed (as shown in Figure 2): high followership prototype and high followership traits (ideal followership), low followership prototype and low followership traits (passable followership), low followership prototype and high followership traits (proactive followership), and high followership prototype and low followership traits (reactive followership).

COR theory further indicates that an individual’s resources will affect their behaviors and performance (Hobfoll et al., 2018). Specifically, followers with sufficient resources are more inclined to initiate taking charge. However, followers who find it hard to access work resources or are threatened with resource loss tend to avoid taking charge. Previous studies have revealed that taking charge will affect an individual’s job performance (Thomas et al., 2010). In summary, based on COR theory, this study explores how followership prototype–traits fit affects followers’ taking charge and then affects their job performance. The proposed hypotheses are described in detail in the next section.

### 2.2 The impact of followership prototype–traits fit on follower’s taking charge

We argue that followership prototype–traits fit more likely stimulates a follower’s taking charge than misfit. Specifically, a follower becomes the leader’s “ideal” follower when the followership traits fit the prototype, which helps the acquisition of instrumental and emotional resources from leaders (Sy, 2010; Wang and Peng, 2016). Regarding instrumental resources, followership prototype–traits fit will increase the leader’s willingness to provide the follower with more work-related information and guidance (Zhang et al., 2012; Peng and Wang, 2018). As for emotional resources, leaders are likelier to trust and support those followers who fit their prototype (van Gils et al., 2010; Peng and Wang, 2018). Previous studies have revealed that followers with more instrumental and emotional resources are more likely to initiate proactive behaviors such as taking charge (Fuller et al., 2006; Halbesleben et al., 2014; Song et al., 2023).

In contrast, followers will find it hard to gain instrumental and emotional resources from leaders when their traits do not fit their prototypes. Thus, such followers are less likely to invest their rare resources to engage in taking charge. We will detail the reasons from two aspects. On the one hand, when a follower’s actual traits do not satisfy the leader’s high expectations, the follower will be identified as a reactive follower (Epitropaki et al., 2013). This type of follower will induce the leader’s negative evaluations and treatments and thus fail to obtain resources from the leader. On the other hand, when a follower’s actual traits are above the leader’s requirements and expectations, the follower will be identified as a proactive follower (Epitropaki et al., 2013). However, this type of follower may also receive adverse treatment from leaders. Prior studies found that proactive followers who go beyond the leader’s expectations could be regarded as a threat to the leader’s status, resulting in fewer resources from the leader (Khan et al., 2018). Thus, reactive and proactive followers struggle to gain work resources and are threatened with resource loss, constraining them from taking charge. Therefore, we hypothesize the following:

*H1*: Compared to a misfit, followers will engage in more taking charge when followership prototype and traits fit.

Followership prototype–traits fit can be fit at high or low levels. We predict that the former type (ideal followership) is more likely to take charge than the latter (passable followership). According to the resource caravan principle of COR theory, an individual’s internal and external resources constitute the total resource pack (Hobfoll, 2011). In our study, ideal followers not only have sufficient positive characteristics such as industriousness and enthusiasm but also can obtain resources from their leaders (van Gils et al., 2010; Uhl-Bien et al., 2014), enabling them to conduct taking charge in the workplace.

In contrast, passable followers need better positive followership traits and behaviors and receive lower expectations from their leaders. Although their weak ability or other mediocre traits are enough to meet leaders’ low expectations, mediocrity will result in difficulties obtaining resources from their leaders (Peng and Wang, 2018). According to the primacy of loss principle from COR theory, passable followers tend to refrain from taking charge to avoid the loss of resources. In addition, passable followers do not have ambitious and high goals at work. Compared with taking risks to initiate proactive behaviors, they tend to avoid mistakes (Xu et al., 2023). Therefore, we hypothesize the following:

*H2*: Followers will take charge more when prototype–traits fit occurs at higher levels rather than at lower levels.

Followership prototype–traits misfit can divide into reactive and proactive followership. We predict that a proactive follower is more likely to take charge than a reactive one. The reasons are detailed in two aspects. On the one hand, proactive followers have more excellent followership traits than their reactive counterparts. Industrious, enthusiastic, and good-citizen followers tend to have the motivation and ability to take charge even without resource support from their leaders (Matthews et al., 2021). In contrast, due to reactive followers failing to meet leaders’ expectations, the leaders will be cautious in providing resources to them (Peng and Wang, 2018; Stegmann et al., 2020). In such cases, reactive followers lack good followership traits and find it difficult to obtain resources from their leaders, thus leading them to avoid taking charge to prevent continuous resource loss.

On the other hand, proactive and reactive followers have different attitudes toward striving for resources. Specifically, proactive followers hold a more positive cognition and affect toward their follower role, which is conducive to taking the initiative (Sy, 2010). In other words, although the leaders may regard proactive followers as a threat and thus retain providing them resources, they also tend to work harder and engage in citizenship behaviors toward organizations out of their heart. Reactive followers may not fulfill their leaders’ expectations due to insufficient abilities and other traits. After weighing the pros and cons, leaders might hesitate to delegate instrumental and emotional resources to reactive followers and even resort to abusive supervision (Khan et al., 2018). In such conditions, reactive followers may decrease the display of risky and extra-role behaviors. Therefore, we hypothesize the following:

*H3*: Compared to followership traits lower than the followership prototype, followers will take charge more when followership traits are higher than the followership prototype.

### 2.3 The mediating role of follower’s taking charge

According to the COR theory, the quantity of individual resources will affect their behaviors and subsequently job performance (Hobfoll et al., 2018). As described in Hypothesis 1, a follower whose followership traits align with the leader’s prototype can acquire more instrumental and emotional resources from the leader (Sy, 2010; Wang and Peng, 2016). With sufficient resources, the follower is more likely to invest valuable resources in initiating proactive behaviors, such as taking charge, to make constructive contributions, or vice versa (Hobfoll, 2001; Fuller et al., 2006). Therefore, we expect that followership prototype–traits fit will impact followers’ job performance via taking charge.

Taking charge involves initiating changes to improve work methods and procedures (Xu et al., 2023). Previous studies have demonstrated that individuals willing to take charge are typically more inclined to invest time and energy to enhance their work, resulting in improved job performance (e.g., Kim et al., 2015; Xu et al., 2023). Additionally, taking charge is characterized by making efforts to initiate change and aims to improve the effectiveness of the organization (Morrison and Phelps, 1999). This implies that followers who initiate taking charge must be willing to invest extra time and energy in their work, which is essential for achieving better job performance (Kim et al., 2015). Previous studies also provided empirical evidence for the relationship between taking charge and job performance. For example, a meta-analysis of proactivity found a significant and positive relationship between taking charge and employees’ job performance (Thomas et al., 2010).

Considering that we have hypothesized the effects of followership prototype–traits fit on taking charge (i.e., Hypothesis 1) and the established positive relationship between taking charge and followers’ job performance, we hypothesize a mediating role for taking charge. Specifically, followership prototype–traits fit has a positive indirect effect on followers’ job performance via taking charge.

Moreover, followership prototype–traits fit affects the number of resources followers can access at work. According to COR theory, followers with more resources are more likely to engage in work and improve their job performance (Hobfoll et al., 2018). Thus, we acknowledge the possibility of the direct effect of followership prototype–traits fit on followers’ job performance. Taken together, combined with H1 to H3, we hypothesize the following:

*H4*: Taking charge partially mediates the relationship between followership prototype–traits fit and followers’ job performance.

### 2.4 The moderating role of follower’s gender

Society has different expectations for different gender, which will impact males’ and females’ responses to identical external events (Eagly and Karau, 2002; Luksyte et al., 2022). We posit that gender difference may act as critical boundary condition which impacts follower’s behavioral responses to followership prototype–traits fit. According to gender stereotypes and social role theory, male followers are expected to be confident, initiative, and achievement-oriented (Eagly and Karau, 2002; Eagly et al., 2020). Male followers will internalize those social role expectations and adopt behaviors that align with the male stereotype to cope with the external environment (Eagly et al., 2020). In comparison, female followers are expected to be mild, inclusive, and relationship-oriented (Eagly and Karau, 2002; Eagly et al., 2020). This social role expectation leads female followers to prioritize getting along with others and obtaining social support during the followership (Luksyte et al., 2022). As a result, female followers tend to allocate more resources toward building relationships with other members of their organization than initiating changes that could challenge authority or lead to interpersonal conflicts (Luksyte et al., 2022). Given that taking charge is characterized by proactive and rule-breaking, it fits with social expectations for male followers. Therefore, compared with their female counterpart, male followers tend to take the initiative when confronted with prototype–traits fit.

Furthermore, social role theory also posits that male follower strives to establish a competent impression and status in the workplace (Luksyte et al., 2022). Xu et al. (2023) found that taking charge signals competence and will also help enhance status. As a result, when male followers find their followership traits fit with leaders’ prototypes, they are more likely to invest resources to engage in taking charge compared with their female counterparts. Thus, we hypothesize the following:

*H5*: Gender moderates the relationship between prototype–traits fit and taking charge. Compared with female followers, the relationship will be stronger for male followers.

### 2.5 Research overview

We use a time-lagged survey (Study 1) and a scenario-based experiment (Study 2) to collectively test the theoretical model. Study 1 is a time-lagged field survey with a multi-wave and multi-source research design to test the overall research model. Study 2 tests the causal relationship between followership prototype–traits fit and followers’ taking charge (H1-H3). This research design helps improve the internal and external validity of our research (Baer et al., 2021).

## 3 Study 1: a time-lagged field survey

### 3.1 Sample and procedure

We collected two waves of multi-source data on-site in 2021 from 14 companies in China. We used random sampling in each department to avoid the interruptions to company’s day-to-day operations. Specifically, we first informed all employees about our study and included a cover letter emphasizing that the responses would be voluntary and confidential. A total of 386 followers and their direct leaders from 133 teams agreed to participate in our study. Two waves of data collection were conducted. Each wave was separated by 3 weeks to alleviate common method variance (Podsakoff et al., 2013). The common job descriptions included human resources, marketing, accounting, and administration.

At Wave 1, 353 followers (91.5% response rate) from 122 teams (91.7% response rate) rated the perception of the direct leader’s followership prototype, their followership traits, and demographic variables. Three weeks later, 262 followers reported taking charge (74.2% response rate), and their 72 direct leaders rated their job performance (59% response rate). The final sample included 262 followers from 72 leaders. Studies have shown that a 3-week temporal separation substantially alleviates common method bias (Ostroff et al., 2002) and erosion effects and that it can capture significant predicted effects (Tsai et al., 2007). We offered a $7 USD-equivalent token per survey completion.

In the final follower sample, 46.2% were women and 52.3% were above 30 years old, and 82.4% had obtained a bachelor’s degree. 96.6% had a 1-year or above organizational tenure with a mean dyadic tenure with the current direct leader of 2.97 (*SD* = 2.75). In the final leader sample, 26.0% were women and 69.0% were above 30 years old, and 92.4% had attained a bachelor’s degree. 50.8% had a 10-year or above organizational tenure.

### 3.2 Measures

As all measures have been established in English, we followed Brislin (1986) translation and back-translation procedures to translate them into Chinese. Unless otherwise noted, a seven-point Likert-type scale (1 = *strongly disagree*, 7 = *strongly agree*) was adopted.

Followership prototype (Cronbach’s *α* = 0.91). We used the nine-item scale from Sy (2010) to rate followers’ perception of a leader’s followership prototype. Nine items such as “hardworking,” “outgoing,” and “reliable” are used to describe the characteristics and behaviors of the leader who think the followers should be. Thus, leader followership prototype represents the environmental demands for followers. Followers’ followership traits reflect the actual abilities exhibited by followers during the followership process (Sy, 2010). Thus, the fit between leaders’ followership prototype and followers’ followership traits aligns with the demand-ability fit (i.e., D-A fit; Kristof-Brown and Guay, 2011). Edwards (1996) pointed out that “*the core mechanism underlying D-A fit is the cognitive comparison of perceived environmental demands to the person’s abilities to meet those demands*” (p. 297). In other words, followers are influenced by the subjective rather than objective perception of their leaders’ followership prototype and their own followership traits. Thus, we invited followers to assess their perceived leaders’ followership prototype.

Followership traits (Cronbach’s α = 0.91). Following Epitropaki and Martin (2005) and Peng and Wang (2018), we used the nine-item scale adapted from Sy (2010) to rate actual characteristics and behaviors as a follower.

Taking charge (Cronbach’s α = 0.89). We used a 10-item scale from Morrison and Phelps (1999) to measure taking charge. Sample item included “I often try to correct a faulty procedure or practice” (1 = *never*, 7 = *always*).

Job performance (Cronbach’s α = 0.93). Direct leaders rated job performance with a three-item scale from Farh et al. (1991). Sample items included “This follower has an outstanding quality of work.”

Controls. Following the recommendations about minimizing the use of control variables in the recent organizational behavior field (Spector and Brannick, 2011; Becker et al., 2016), we only controlled for theoretically meaningful controls that could influence the associations between the key studied variables. Existing research on the relationship between leader–follower congruence and job performance has found that variables such as age similarity, education similarity, and dyadic tenure can influence the causal relationships among the studied variables (e.g., Zhang et al., 2012; Carter and Mossholder, 2015; Tepper et al., 2018). For example, Zhang et al. (2012) discovered that demographic congruence between leaders and followers can impact the relationship between leader–follower congruence on personality and followers’ job performance. In line with these studies, we also controlled for them. At the team level, we controlled for team size and team age to exclude possible alternative explanations (Liu et al., 2021).

### 3.3 Analytical strategy

*Multilevel polynomial regressions*. To account for the nested structure of our data (262 followers nested in 72 leaders), we employed the ICC1 approach to examine the appropriateness of multilevel analyses. The results indicated that team membership accounted for a significant variance in job performance (*ICC1* = 0.16, *F-*statistics = 1.72, *p* < 0.01). Therefore, we conducted two-level polynomial regression and response surface with the HLM 6.08 software to test our theoretical model (Edwards and Parry, 1993; Edwards and Cable, 2009). Specifically, we used the leader’s followership prototype (*L*) and the follower’s followership traits (*F*) first-order terms to form three second-order terms (*L*^2^, *F*^2^, and *L × F*). These five polynomial terms were then used to examine the fit/misfit effects between *L* and *F*. Prior to calculating the second-order terms, we standardized first-order terms (*L* and *F*) to reduce the effect of multicollinearity (Zhang et al., 2012). We then examined the followership prototype–traits fit/misfit effects by estimating the following equation:


$$\begin{array}{l} \text{Taking}\;\text{charge}=b_{0}+b_{01}\;\text{Team}\;\text{size}+b_{02}\;\text{Team}\;\text{age}\\ +b_{03}\;\text{Age}\;\text{dissimilarity}+b_{04}\;\text{Education}\;\text{dissimilarity}\\ +b_{05}\;\text{Dyadic}\;\text{tenure}+b_{1}\;L+b_{2}\;F+b_{3}\;L^{2}+b_{4}\;L\times F\\ +b_{5}\;F^{2} \end{array}$$

Additionally, the polynomial regression coefficients (*b_0_–b_5_*) were utilized to plot the three-dimensional response surface, in which *L* and *F* values run along the *X*-axis and *Y*-axis of the horizontal plane. The followers’ taking charge was plotted on the vertical axis (*Z*-axis).

To examine the effects of followership prototype–traits fit and misfit (H1), we evaluated the curvature along the misfit line (*b_3_* – *b_4_* + *b_5_*; Edwards and Cable, 2009). A positive curvature would indicate that the follower’s taking charge is higher when *L* and *F* are fit in comparison with *L* and *F* are misfit, in support to H1.

To examine the fit effect at high and low levels (H2), we calculate the slope along the fit line (*b*_1_ + *b*_2_). A positive and significant slope would indicate that the fit at high levels leads to higher follower’s taking charge than at low levels, in supporting to H2.

To examine the asymmetry of the misfit effect (H3), we calculated the lateral shift quantity [(*b*_2_ – *b*_1_)]/[2× (*b*_3_ – *b*_4_ + *b*_5_)] of the response surface along the misfit line. According to Cole et al. (2013), the lateral shift quantity reflects the magnitude and direction of a lateral shift. For the convex surface, a negative value represents a lateral shift toward the region where the followership traits are greater than the followership prototype (*L* < *F*). This means the follower’s taking charge increases sharper in the *L* < *F* region than in the *L* > *F* region, thus supporting H3. Given that the lateral shift quantity is a non-linear combination of regression coefficients, we tested its significance by utilizing 20,000 bootstrapped samples with *RStudio* to estimate 95% CIs.

*Mediation test using the block* var*iable approach*. To test the indirect effect of followership prototype–traits fit/misfit on followers’ job performance via taking charge (H4), we employed the block variable approach outlined by Edwards and Cable (2009). We first created a block variable to represent followership prototype–traits fit/misfit effect of the five polynomial regression terms (*L*, *F*, *L^2^*, *L* × *F*, and *F^2^*) with a weighted linear composite. The respective weights are the estimated regression coefficients in the polynomial regression. We then regressed the polynomial regression model again to obtain (1) the path coefficient “a” for the effect of followership prototype–traits fit/misfit on taking charge; (2) the path coefficient “b” for the effect of taking charge on job performance by controlling for the effects of followership prototype–traits fit/misfit; and (3) the path coefficient “c” for the effect of the followership prototype–traits fit/misfit on job performance after controlling for the effects of taking charge. Finally, we calculated the indirect effects (i.e., a × b) and tested its significance with the *bootstrapping* approach.

*Moderation test using the Chow test*. To test the moderating role of followers’ gender on the relationship between followership prototype–traits fit/misfit effect on followers’ taking charge (H5), we employed the Chow test outlined by Lee and Antonakis (2014). Specifically, we split our sample into male and female samples and conducted multilevel polynomial regression on the two samples. We then compare the curvature difference (*b3* – *b4* + *b5*) along the misfit line (*L = –F*). When the difference is significant, the H5 was thus supported.

### 3.4 Results

Table 1 reports the descriptive statistics of all studied variables and the correlation matrix in Study 1. Given the high correlation between the followership prototype and followership traits, we followed Song and Chen (2021) approach proposed by Edwards to establish that these two measures are empirically distinct. Specifically, we calculated that the correlations (*r_j_*) between their respective factors are significantly less than unity. To test it, we used the standard error of the correlation to construct a 95% confidence interval (CI) and assess whether it excludes 1. The results showed that *r*_Industry_ = 0.67, 95% CI = [0.58, 0.75]; *r*_Enthusiasm_ = 0.62, 95% CI = [0.55, 0.70]; *r*_Good-citizen_ = 0.64, 95% CI = [0.55, 0.72], excluding 1. In addition, we also evaluate whether the factor correlation is not exclusive. Testing whether the outcome is maximized when the prototype and traits are equal will be difficult if they are primarily on one side. We inspected the bivariate scatterplot of the prototype and traits scores and found that the cases in our data are distributed on both sides of the *Y* = *X* line, illustrating the appropriateness of exploring their fit/misfit effect.

**Table 1:** Means, standard deviations, and correlation coefficients (Study 1).

Variables	1	2	3	4	5	6	7	8	9	10
1. Team size	—									
2. Team age	0.44^**^	—								
3. Age dissimilarity^a^	0.06	0.13^*^	—							
4. Education dissimilarity^b^	−0.13^*^	−0.06	−0.02	—						
5. Dyadic tenure	0.18^**^	0.22^**^	0.07	−0.02	—					
6. Followership prototype	0.05	−0.03	−0.01	0.04	−0.16^*^	*(0.91)*				
7. Followership trait	0.04	−0.03	−0.03	0.01	−0.15^*^	0.72^**^	*(0.91)*			
8. Taking charge	0.14^*^	0.05	−0.01	−0.05	0.05	0.26^**^	0.47^**^	(*0.89*)		
9. Job performance	−0.00	0.10	−0.08	−0.03	0.00	0.23^**^	0.38^**^	0.30^**^	*(0.93)*	
10. Follower gender	−0.08	−0.02	−0.07	−0.01	−0.10	0.16^*^	0.11	−0.02	−0.04	—
*M*	31.52	5.13	0.62	0.56	2.97	6.28	6.14	4.67	5.26	1.46
*SD*	81.78	5.37	0.61	0.59	2.75	0.68	0.64	0.70	0.93	0.50

*N* = 72 at the team level and *N* = 262 at the individual level. ^a^1 = less than 20 years; 2 = 21–30 years; 3 = 31–40 years; 4 = 41–50 years; 5 = more than 51 years; ^b^1 = high school or below; 2 = associate’s degree; 3 = bachelor’s degree; 4 = master’s degree; 5 = doctorate. Cronbach’s α values are in parentheses on the diagonal. ^*^p < 0.05 and ^**^p < 0.01.

We conducted confirmatory factor analyses to assess the distinctiveness of our constructs. The results showed that our four-factor model yielded a satisfactory fit (*χ*^2^_(417)_ = 821.78, *p* < 0.001; CFI = 0.92, TLI = 0.92, RMSEA = 0.06, SRMR = 0.05) and was significantly better to all other alternative models (all *p*s [Δχ^2^] < 0.001).

We conducted two preliminary analyses before hypothesis testing. As shown in Table 2, the second-order polynomial terms account for substantial incremental variance in followers’ taking charge [*F*_(3, 251)_ = 8.23, *p* < 0.001], which allows for examining a non-linear impact of followership prototype and traits on taking charge (Edwards, 2002). In addition, according to Edwards and Parry (1993), the first principal axis of the response surface (*F* = *p*_10_ + *p*_11_*L*) cannot be shifted laterally (*p*_10_ including 0) or reversed (*p*_11_ including −1) along the misfit line if the minimum value of taking charge located on the misfit line (*L* = *−F*). Table 2 denotes that the slope (*p*_11_) did not significantly differ from −1 (95% CI [−1.11, −0.62]), and the intercept (*p*_10_) did not significantly differ from 0 (95% CI [*−*1.84, 0.49]).

**Table 2:** Polynomial regression of followership prototype–traits fit on followers’ taking charge (Study 1).

Variables	Taking charge			
	Model_1_		Model_2_	
	*b*	*SE*	*b*	*SE*
*b*_0_ Intercepts	4.67^***^	0.04	4.74^***^	0.05
Controls				
*b*_01_ Team size	0.06	0.03	0.05	0.03
*b*_02_ Team age	0.01	0.05	0.00	0.05
*b*_03_ Age dissimilarity	−0.01	0.03	0.01	0.03
*b*_04_ Education dissimilarity	−0.04	0.04	−0.04	0.04
*b*_05_ Dyadic tenure	0.06^*^	0.03	0.05^*^	0.03
Response surface terms				
*b*_1_ Followership prototype (*L*)	−0.16^**^	0.05	−0.13^*^	0.06
*b*_2_ Followership traits (*F*)	0.41^***^	0.05	0.36^***^	0.05
*b*_3_ *L*^2^			−0.24^***^	0.04
*b*_4_ *L* × *F*			0.45^***^	0.09
*b*_5_ *F*^2^			−0.19^**^	0.06
*F*-*Statistics* for the 3 quadratic terms (*L*^2^, *L* × *F*, *F*^2^)			8.23^***^	
*R* ^2^	0.33^***^		0.39^***^	
Δ*R*^2^			0.06^***^	
Response surface analyses				
Stationary point (*L*_0_, *F*_0_)			(−4.82, −4.84)	
The first principal axis *F* = *p*_10_ + *p*_11_*L*			*F* = −0.56 –0.89 *L*	
The lateral shift quantity (*b_2_ –b_1_*)/[2 × (*b_3_ –b_4_+ b_5_*)]			−0.29^***^	
Fit line (*L* = *F*)				
Slope (*b*_1_ + *b*_2_)			0.23^**^	0.08
Curvature (*b*_3_ + *b*_4_ + *b*_5_)			0.02	0.12
Misfit line (*L* = –*F*)				
Slope (*b*_1_–*b*_2_)			−0.48^***^	0.08
Curvature (*b*_3_–*b*_4_ + *b*_5_)			−0.88^***^	0.12

*N* = 72 at the team level and *N* = 262 at the individual level. Values represent unstandardized coefficients. ^*^p < 0.05, ^**^p < 0.01, and ^***^p < 0.001.

**Figure 3:**
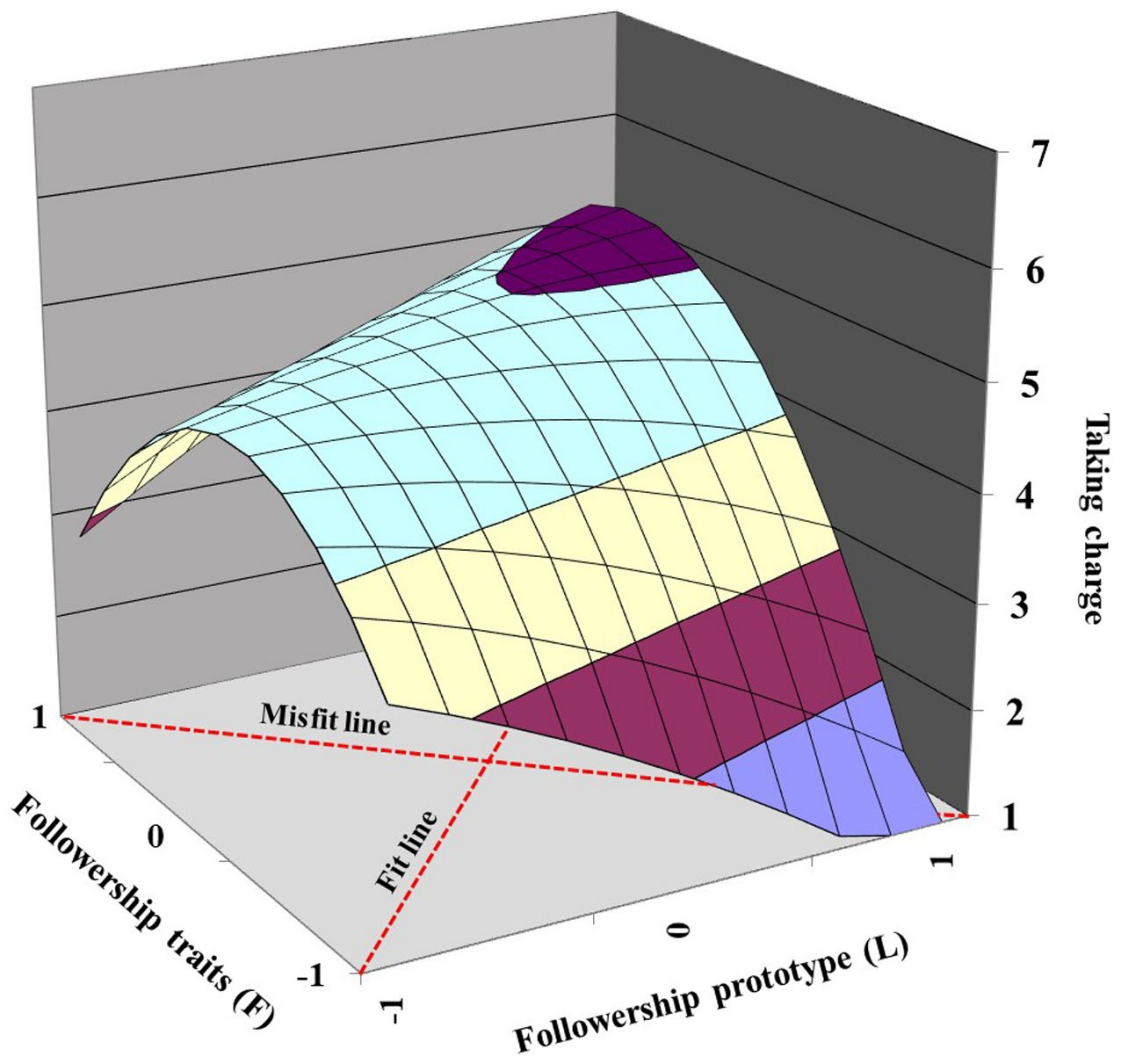
Impact of followership prototype–traits fit on taking charge (Study 1).

H1 predicted a fit effect of followership prototype and traits on followers taking charge. As shown in Table 2, the curvature (*b_3_* – *b_4_* + *b_5_*) along the misfit line (*L* = −*F*) was significantly negative (*curvature = −*0.88, *p* < 0.001). The response surface shown in Figure 3 indicated an inverted U-shaped curve along the misfit line (*L* = −*F*). It indicated that followers’ taking charge is higher when the followership prototype fits with followership traits, and any deviations from the fit line decrease taking charge, in supporting H1.

H2 predicted that followers’ taking charge is higher when followership prototype–traits fit at a high level compared with a low level. Table 2 shows that the slope (*b_1_* + *b_2_*) along the fit line (*L* = *F*) was significantly positive (0.23, *p* = 0.004). The response surface shown in Figure 3 also indicates that the follower’s taking charge is higher at the rear corner (*L* = *F =* 1) than at the front corner (*L* = *F* = −1). Thus, H2 was supported.

H3 predicted that taking charge is higher when followership traits are higher than the followership prototype compared to followership traits lower than the followership prototype. Table 2 presents the lateral shift quantity as negative and significant (−0.29, 95% CI = [−0.52, −0.14]), indicating a shift toward the region where *F* is greater than *L*. In addition, Figure 3 presents this asymmetrical effect, in which taking charge was higher at the left corner (*L* < *F*) than at the right corner (*L* > *F*). Thus, H3 was supported.

H4 predicted the partial mediating effect of taking charge in the relationship between followership prototype–traits fit and job performance. Table 3 shows that the direct effect of followership prototype–traits fit on job performance was significant (*b* = 0.80, *p* < 0.01). The followership prototype–traits fit was significantly and positively associated with taking charge (*b* = 1.01, *p* < 0.001). Taking charge also significantly and positively affected job performance (*b* = 0.25, *p* < 0.001). Furthermore, the indirect effect of the followership prototype–traits fit on job performance through taking charge was significant (*indirect effect* = 0.26, 95% CI [0.09, 0.44]). Taking both the direct and indirect effects into consideration, H4 was supported.

**Table 3:** Results of path coefficient regression (Study 1).

Variables	Mediator	Outcome
	Taking charge	Job performance
Controls		
Team size	0.05 (0.03)	−0.13 (0.04)
Team age	0.01 (0.05)	0.16 (0.07)
Age dissimilarity	−0.02 (0.03)	−0.08 (0.07)
Education dissimilarity	−0.04 (0.04)	−0.04 (0.05)
Dyadic tenure	0.07^*^ (0.03)	0.01 (0.05)
Independence variable		
Block variable (Followership prototype–traits fit)	1.01^***^ (0.12)	0.80^**^ (0.17)
Mediator variable		
Taking charge		0.25^***^ (0.08)
Indirect effect		
Indirect path	Effect size	95% CI
Followership prototype–traits fit → Taking charge → Job performance	0.26	[0.09, 0.44]

*N* = 72 at the team level and *N* = 262 at the individual level. Values represent unstandardized coefficients. ^*^p < 0.05, ^**^p < 0.01, and ^***^p < 0.001.

**Figure 4:**
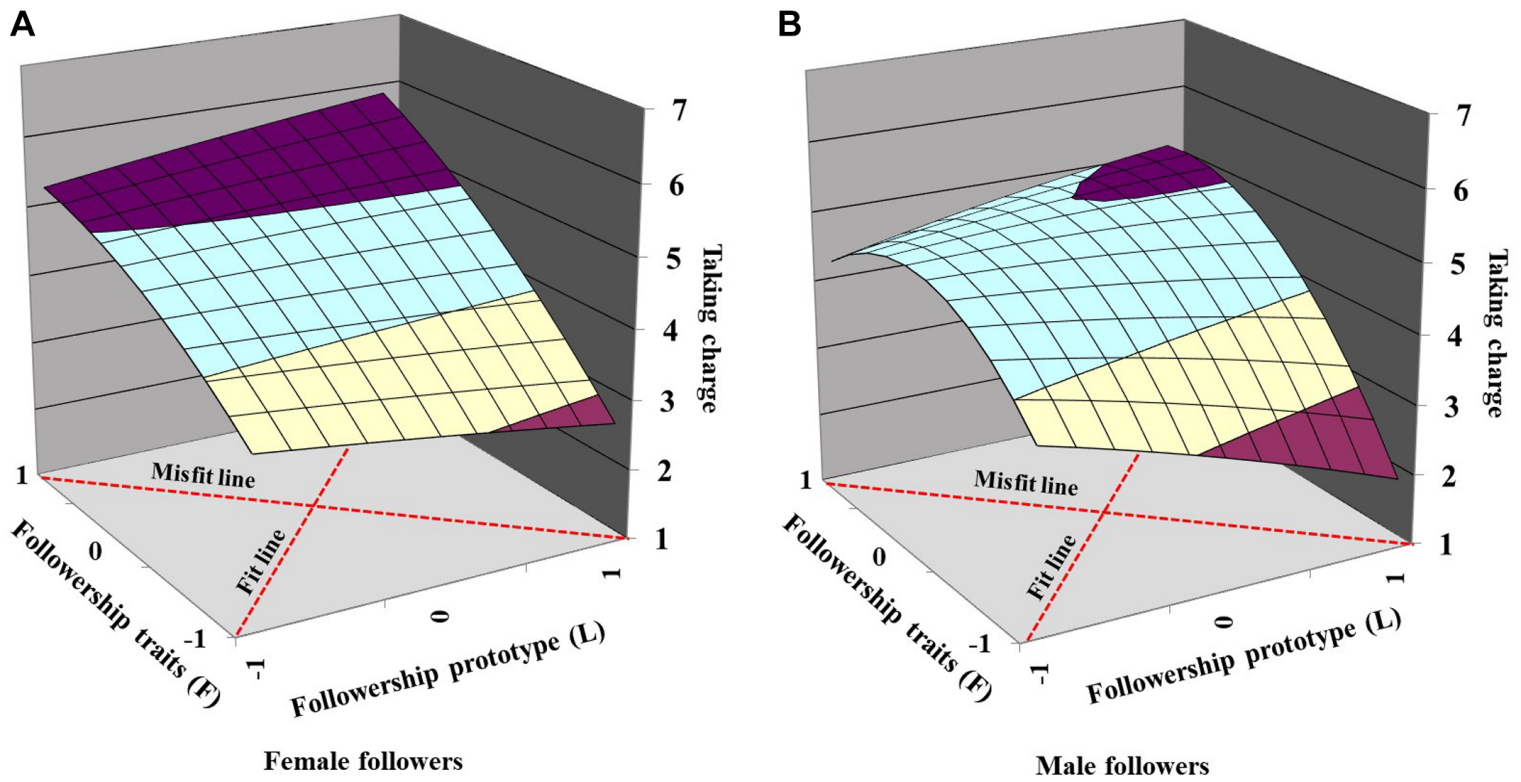
**(A,B)** Effects of followership prototype–traits fit on taking charge for different gender (Study 1).

H5 predicted that the relationship between followership prototype–traits fit and taking charge would be stronger with male followers than female followers. As shown in Table 4, the curvature of the misfit line (*L* = −*F*) was significant in the male follower subsample (*curvature* = *−*0.39, *p* < 0.001) but not significant in the female follower subsample (*curvature* = *−*0.17, *p* = 0.14). This comparison effect is also depicted in Figures 4A,B, in which the surface along the fit line (*L* = *F*) was steeper in male followers than female followers. Additionally, there was a significant difference between the male and female follower samples (*χ*^2^_(2)_ = 13.55, *p* = 0.001) based on the Chow test. Thus, H5 was supported.

**Table 4:** Moderating effect of gender (Study 1).

Variables	Taking charge			
	Male follower		Female follower	
	*b*	*SE*	*b*	*SE*
*b*_0_ Intercepts	4.74^***^	0.07	4.62^***^	0.07
Controls				
*b*_01_ Team size	0.06^*^	0.03	0.03	0.03
*b*_02_ Team age	0.04	0.05	−0.04	0.05
*b*_03_ Age dissimilarity	−0.03	0.05	−0.01	0.06
*b*_04_ Education dissimilarity	−0.10	0.06	0.03	0.05
*b*_05_ Dyadic tenure	0.02	0.05	0.04	0.03
Response surface terms				
*b*_1_ Followership prototype (*L*)	−0.18^*^	0.09	−0.02	0.12
*b*_2_ Followership traits (*F*)	0.32^***^	0.09	0.42^***^	0.11
*b*_3_ *L*^2^	−0.07^**^	0.03	−0.02	0.05
*b*_4_ *L* × *F*	0.21	0.12	0.11	0.13
*b*_5_ *F*^2^	−0.11	0.07	−0.03	0.09
*R*^2^	0.22		0.35	
Fit line (*L* = *F*)				
Slope (*b*_1_ + *b*_2_)	0.14	0.07	0.40	0.22
Curvature (*b*_3_ + *b*_4_ + *b*_5_)	0.03	0.06	0.06	0.03
Misfit line (*L* = –*F*)				
Slope (*b*_1_–*b*_2_)	−0.50^**^	0.16	−0.44^***^	0.06
Curvature (*b*_3_–*b*_4_ + *b*_5_)	−0.39^***^	0.10	−0.17	0.11
Coefficient comparison	*χ*^2^_(2)_ = 13.55^**^			

*N* = 72 at the team level and *N* = 262 at the individual level. Values represent unstandardized coefficients. ^*^p < 0.05, ^**^p < 0.01, and ^***^p < 0.001.

We also conducted post-hoc analyses to examine the direct effect of followership prototype–traits fit on job performance. The results indicated that the curvature (*b_3_* – *b_4_* + *b_5_*) along the misfit line (*L* = −*F*) was negative but non-significant (*curvature* = −0.13, *n.s.*). It indicated that compared with a misfit, followers’ job performance was slightly higher when followership prototype and traits fit. The slope (*b_1_* + *b_2_*) along the fit line (*L* = *F*) was significantly positive (*slope* = 0.33, *p* < 0.01). Thus, followers’ job performance was significantly higher when prototype–traits fit occurs at higher levels rather than at lower levels. Additionally, the lateral shift quantity was negative and significant (*lateral shift quantity* = −2.11, *p* < 0.05). Therefore, compared with followership traits lower than the followership prototype, followers’ job performance was higher when followership traits were higher than the followership prototype. As we can see, the influence patterns of prototype–traits fit on follower taking charge were similar to those on follower job performance.

### 3.5 Discussion of study 1 findings

The results of Study 1 showed that compared with the misfit, followership prototype–traits fit is more likely to stimulate followers’ taking charge. Among the fit condition, high vs. low levels of followership prototype–traits fit is more likely to stimulate followers’ taking charge. Among the misfit condition, low followership prototype and high followership traits condition is more likely to stimulate followers’ taking charge compared with high followership prototype and low followership traits condition. Furthermore, followers’ taking charge partially mediates the impact of the followership prototype–traits fit on followers’ job performance. Additionally, the impact of followership prototype–traits fit on followers’ taking charge is more salient for male followers than for female followers.

While this study has notable strengths, such as its strong external validity and multi-wave, multiple-source research design to alleviate common method bias (Gabriel et al., 2019), it is not without limitations. Specifically, in spite of using temporal separation in our data collection, the survey design has difficulty in causal inferences. To address the concern, we conducted Study 2 by creating a 2 (followership prototypes: high vs. low) × 2 (followership traits: high vs. low) between-subjects factorial design to establish the causal relationship between the focal variables (e.g., Podsakoff et al., 2013). The combination of a survey and an experiment helps improve the internal and external validity of our study.

## 4 Study 2: a scenario-based experiment

### 4.1 Preliminary study

We employed a preliminary study to examine the effectiveness of our manipulation materials of followership prototypes and followership traits. Specifically, we first prepared manipulation materials based on the measures developed by Sy (2010). We then invited two professors and nine Ph.D. candidates who majored in business administration to polish the language and reach a consensus on the wording. Lastly, we recruited 159 employees to test the manipulations of followership prototypes and 187 to test the manipulations of followership traits through an online data collection website wjx.cn (an online survey platform similar to MTurk), respectively.

The participants were randomly assigned to four conditions, namely, high−/low-level followership prototypes and high−/low-level followership traits. They were asked to complete the surveys about the leader’s followership prototype and followership traits after reading the assigned manipulation materials. The mean values for the high (*M* = 4.22, *SD* = 0.43) and low (*M* = 3.19, *SD* = 1.01) followership prototype conditions significantly differed [*t* (157) = 8.79, *p* < 0.001, Cohen’s *d* = 1.41]. The mean values for the high (*M* = 4.30, *SD* = 0.44) and low (*M* = 2.95, *SD* = 1.03) followership traits conditions significantly differed [*t* (185) = 11.78, *p* < 0.001, Cohen’s *d* = 1.71]. The manipulations for both followership prototype and followership traits were thus effective.

### 4.2 Scenario-based experiment

#### 4.2.1 Samples and procedure

We invited 160 undergraduates majoring in business administration at a university in China to participate in a scenario-based experiment in 2022. The students participated voluntarily and were reimbursed with a gift. Among the participants, 69.4% were female follower with an average participant age of 18.76 (*SD* = 0.95) years old. Each group had 40 participants.

We adopted a 2 (followership prototypes: high vs. low) × 2 (followership traits: high vs. low) between-subjects factorial design. The participating students were gathered in a classroom to read the materials and fill out the questionnaires. In the scenario experiment, each participant was randomly assigned to four conditions. The manipulation materials came from the preliminary study. The experiment was conducted with the following steps. The participants first reported their demographics and were then asked to read the manipulation materials corresponding to their assigned condition. They then completed a manipulation check and assessed their own levels of taking charge. To assess their awareness of the manipulation, we asked the participants to briefly describe the purpose of the experiment (Grégoire et al., 2019). None gave a correct response. We finally thanked and debriefed all of the participants.

Followership prototype manipulation. The manipulation scripts in the high/low followership prototype condition are “You have been working with your direct supervisor for 2 years. You think in his mind, the ideal follower needs/not needs to be hardworking, productive, goes above and beyond, excited, outgoing, happy, loyal, reliable, and a good team player.”

Followership traits manipulation. The manipulation scripts in the high/low followership traits condition are: “As a follower, you are always/rarely hardworking, productive, goes above beyond, excited, outgoing, happy, loyal, reliable, and a good team player.”

#### 4.2.2 Measures

The measures in Study 2 were the same as those in study 1. All items were measured with a five-point Likert-type scale (1 = *extremely low*, 5 = *extremely high*) unless otherwise noted.

The Cronbach’s α values for followership prototype, followership traits, and taking charge (1 = *never*, 5 = *always*) are 0.96, 0.98, and 0.94, respectively.

#### 4.2.3 Manipulation checks

An *independent samples t*-*test* was used to conduct the manipulation check. The results showed that the mean values for the high (*M* = 4.13, *SD* = 0.46) and low (*M* = 2.52, *SD* = 0.74) followership prototype conditions significantly differed [*t*(158) = 16.40, *p* < 0.001, Cohen’s *d* = 2.61]. Meanwhile, the mean values for the high (*M* = 4.40, *SD* = 0.41) and low (*M* = 2.50, *SD* = 0.62) followership traits conditions significantly differed [*t*(158) = 22.88, *p* < 0.001, Cohen’s *d* = 3.61]. The interventions were thus effective.

#### 4.2.4 Hypothesis testing

**Figure 5:**
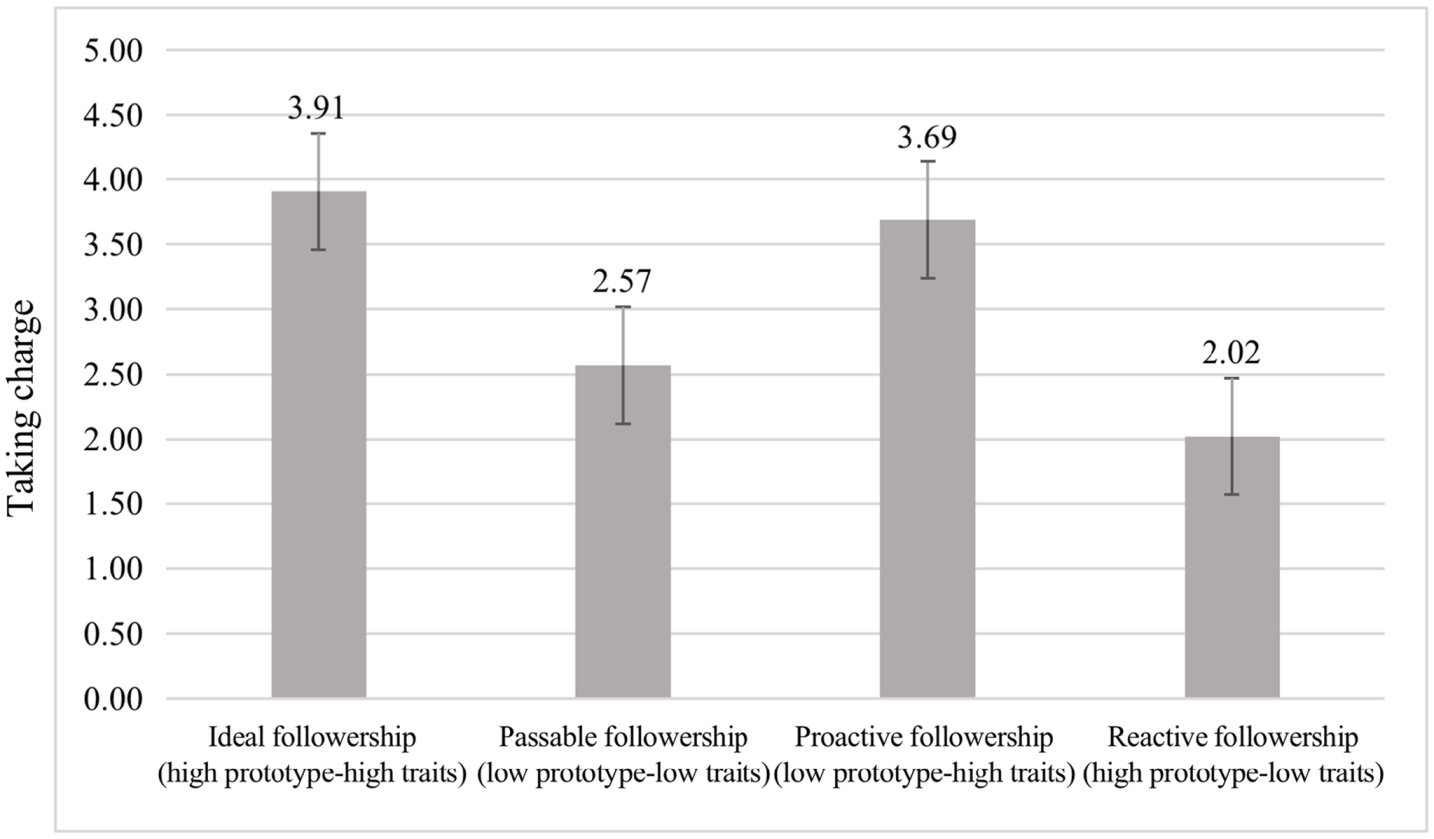
Means across experimental conditions for taking charge (Study 2). Prototype represents leaders’ followership prototype; traits represent followers’ followership traits.

Table 5 shows the descriptive statistics and correlations for the studied variables in Study 2. We tested H1–H3 by independent samples *t*-test and post-hoc pairwise comparisons using the *Sidak* adjustment. The means of each condition are shown in Table 6 and depicted in Figure 5.

**Table 5:** Descriptive statistics (Study 2).

Variables	*M*	*SD*	1	2	3	4	5
1. Gender	0.31	0.46	—				
2. Age	18.76	0.95	0.14	—			
3. Followership prototype	0.50	0.50	0.05	−0.04	*(0.96)*		
4. Followership trait	0.50	0.50	0.11	0.04	0.00	*(0.98)*	
5. Taking charge	3.05	0.94	0.17^*^	−0.01	−0.09	0.80^**^	*(0.94)*

*N* = 160. Gender: 1 = male; 0 = female. Cronbach’s α values are in parentheses on the diagonal. ^*^p < 0.05 and ^**^p < 0.01.

**Table 6:** Means across conditions and mean differences among conditions of taking charge (Study 2).

		H1: Mean difference		H2: Mean difference	H3: Mean difference
		Vs. High prototype–Low traits	Vs. Low prototype–High traits	Vs. Low prototype–Low traits	Vs. High prototype–Low traits
Fit	High prototype–High traits	1.89^***^	0.22	1.35^***^	––
	Low prototype–Low traits	0.54^***^	−1.13^***^	––	––
Misfit	High prototype–Low traits	––	––	––	––
	Low prototype–High traits	––	––	––	1.67^***^

*N* = 160. Prototype represents leaders’ followership prototype; traits represents followers’ followership traits. ^***^p < 0.001.

As shown in Table 6 and Figure 5, the participants in fit conditions—both at high and low levels—reported higher taking charge (3.24), in comparison with conditions of misfit (2.86). The mean difference for these two groups was 0.38 (*p* < 0.01), supporting H1. In the fit group, participants in the high followership prototype–high followership traits condition reported higher taking charge (3.91) compared to the low followership prototype–low followership traits condition (2.57). The mean difference for the above two conditions was 1.35 (*p* < 0.01), supporting H2. In the case of misfit, participants in the low followership prototype–high followership traits condition reported higher taking charge (3.69), in comparison with the high followership prototype–low followership traits condition (1.67). The mean difference for the above two conditions was 1.67 (*p* < 0.01), supporting H3.

## 5 Discussion

Drawing from COR theory, our study explores *how*, *why*, and *when* followership prototype–traits fit impacts followers’ job performance from the leader–follower dyadic perspective. The results of a time-lagged survey (Study 1) and a scenario-based experiment (Study 2) reveal that (1) compared with the misfit, followership prototype–traits fit is more likely to stimulate followers’ taking charge; (2) compared with low levels of fit, high-level followership prototype–traits fit is more likely to stimulate followers’ taking charge; (3) compared with high followership prototype and low followership traits condition, low followership prototype and high followership traits condition is more likely to stimulate followers’ taking charge; (4) followers’ taking charge partially mediates the impact of the followership prototype–traits fit on followers’ job performance; and (5) the impact of followership prototype–traits fit on followers’ taking charge is more salient for male followers than for female followers.

### 5.1 Theoretical implications

Our research makes several notable theoretical contributions. Our research enriches the understanding of the antecedents of followers’ job performance by revealing the impact of followership prototype–traits fit with a leader–follower dyadic perspective. Previous research on factors driving job performance has often been explored from a single perspective, either focusing on leaders or followers. Recently, some scholars have adopted a dyadic perspective by investigating the impact of leader–follower congruence on job performance (Zhang et al., 2012; Carter and Mossholder, 2015). However, these studies have primarily focused on congruence in roles, personality, and values between leaders and followers, neglecting the congruence between followers’ followership prototype and leaders’ followership traits. This study uses a dyadic perspective to examine the influence of followership prototype–traits fit on followers’ job performance. By doing so, we not only help capture the bidirectionality and complexity presented in the leader–follower interaction process during the followership but also obtain a more comprehensive understanding of the impacts of leader–follower congruence on followers’ job performance.

Second, prior studies have mainly examined the effects of followership prototype–traits fit from role theory and job demand-resource model (Wang and Peng, 2016; Peng and Wang, 2018). In contrast, this study takes a new theoretical perspective based on COR theory to investigate how followership prototype–traits fit affects followers’ taking charge via impacting their resource changes. By doing so, we not only clarify how and why followership prototype–traits fit impact follower job performance but also enrich our understanding of the followership process. Furthermore, while most previous prototype–traits fit research has focused on its influence on follower in-role behavior, this study extends its effects to follower extra-role behavior, which helps to enrich our understanding of the causal relationship network of followership prototype–traits fit.

Third, our study enriches and expands the antecedents of taking charge. Previous research on the antecedents of taking charge has mainly focused on leadership and individual differences from a single perspective (e.g., Wu et al., 2018). However, antecedents that solely focus on either leaders or followers are insufficient to clearly understand why taking charge happens. To fill this gap, we consider both the leaders’ followership prototype and followers’ followership traits and explore their comprehensive impact on followers’ taking charge. By doing so, we not only help to deepen our understanding of the driving factors of taking charge but also demonstrate that followers’ taking charge is the function of the interaction between personal characteristics and the external environment, indirectly confirming the classical person–environment interaction lens.

Fourth, although gender differences are prevalent in the followership process, previous followership prototype–follower traits studies have yet to explore the boundary effects of gender. We incorporated gender as a crucial boundary condition and proved that male and female followers have different preferences in employing taking charge when reacting to followership prototype–traits fit. By doing so, we broaden our understanding of the role of gender in the process of followership prototype–traits fits and contribute to our knowledge about the social role theory.

### 5.2 Practical implications

Our study also has some practical implications. First, our study reveals that followership prototype–traits fit significantly influences followers’ job performance via taking charge. To leverage this insight, organizations should consider assessing their members’ followership traits and their leaders’ followership prototypes during the recruitment and selection processes. This information can be used to pair leaders with ideal followers to build a proactive and high-performance work team.

Second, this study finds that followers’ taking charge is higher when followership prototype–traits fit at a high level compared with a low level. This enlightens that the organizations should facilitate high-level followership prototype–traits fit. Specifically, on the one hand, to shape leaders’ high-level prototypes, organizations should emphasize the importance of leaders to form positive followership prototypes in regular meetings and trainings. On the other hand, to shape members’ high-level followership traits, organizations should take measures such as skills training to enhance members’ working abilities, enthusiasm, and moralities.

Third, past research suggests that a leader’s followership prototypes are flexible and can change over time (Epitropaki et al., 2013). This means that if a follower’s traits do not align with a leader’s followership prototype, the leader can adjust their prototype to better suit the follower’s current traits. This adaptation can effectively motivate followers to take charge and become more engaged.

Fourth, we found that the relationship between followership prototype–traits fit and taking charge would be stronger with male followers than with female followers. As such, policymakers can employ differentiated tactics to inspire taking charge behaviors for male and female followers. Specifically, leaders can offer additional material and spiritual incentives for the female follower to encourage them to take the initiative and provide instant feedback throughout the process. Additionally, female employees should take the opportunity to actively take the initiatives to express their excellent work capabilities to their leaders fully.

### 5.3 Limitations and future research directions

Our research has some limitations, which provide several directions for future research. First, previous followership prototype–traits fit studies have mainly focused on its function in stimulating positive attitudes and behaviors (Wang and Peng, 2016; Peng and Wang, 2018), neglecting its function for alleviating negative attitudes and behaviors. Future studies can explore this line of inquiry. Second, we only conducted two-wave data collection in Study 2. Although we conducted a scenario experiment to test the causal relationship between followership prototype–traits fit on taking charge, we did not test the mediating role of taking charge in our study, leading to the possibility of reverse causality. Future research should collect longitudinal panel data for causality inferences (Tepper et al., 2018). Third, we invited followers to evaluate their perceived leaders’ followership prototype (Edwards, 1996). We also acknowledge that conducting research solely based on followers’ perceptions has its limitations. This is particularly evident when followers may not accurately perceive the leader’s expectations for an ideal follower in certain situations, such as when leaders and followers lack communication (Flynn and Lide, 2023). Therefore, we encourage future research to delve into the alignment between the leader’s actual followership prototype and the follower’s followership traits. Fourth, our research draws upon COR theory to examine how followership prototype–traits fit affects followers’ job performance. However, future studies may explore additional mechanisms from appropriate theoretical perspectives. For instance, followership prototype–traits misfit can be regarded as a stressor, and we can draw on the cognitive appraisal theory to analyze how it impacts followers’ behaviors and performance (Song and Chen, 2021). Finally, our study explores the moderating role of follower gender. We encourage future studies to explore other theoretically relevant moderators (e.g., individual personality, leader and follower demographic similarity, or team climate). These explorations can enrich our understanding of the boundary conditions that moderate the influence of leader–follower congruence on work behaviors.

## Data availability statement

The raw data supporting the conclusions of this article will be made available by the authors, without undue reservation.

## Ethics statement

The studies involving humans were approved by School of Business Administration, Southwestern University of Finance and Economics. The studies were conducted in accordance with the local legislation and institutional requirements. The participants provided their written informed consent to participate in this study.

## Author contributions

JY: Conceptualization, Formal analysis, Investigation, Writing – review & editing, Data curation, Methodology. JF: Supervision, Writing – original draft.

## Footnotes

^1^It is worth noting that two existing studies on followership prototype-trait congruence have focused on their effects on benevolent leadership and follower well-being respectively (Wang and Peng, 2016; Peng and Wang, 2018). In contrast, our research focuses on the core work outcome, namely job performance. Therefore, our research differs significantly from previous work on followership prototype-traits congruence.
